# Association Between Vitamin D Receptor Gene Polymorphisms and Polycystic Ovary Syndrome Risk: A Meta-Analysis

**DOI:** 10.3389/fphys.2018.01902

**Published:** 2019-01-10

**Authors:** Yu-Ming Niu, Ya-Dong Wang, Guang-Bin Jiang, Gang Bai, Hong-Bo Chai, Xue-Feng Li, Yuan-Yuan Hu, Ming Shen

**Affiliations:** ^1^Department of Stomatology and Center for Evidence-Based Medicine and Clinical Research, Taihe Hospital, Hubei University of Medicine, Shiyan, China; ^2^Department of Oral and Maxillary Surgery, Gui Zhou Provincial People's Hospital, Guiyang, China; ^3^Department of Radiology, Suizhou Central Hospital, Suizhou, China; ^4^Department of Medical Ultrasound, Taihe Hospital, Hubei University of Medicine, Shiyan, China; ^5^Department of Endocrinology, Taihe Hospital, Hubei University of Medicine, Shiyan, China; ^6^Jiangsu Key Laboratory of Oral Diseases, Department of Dental Implant, Affiliated Hospital of Stomatology, Nanjing Medical University, Nanjing, China

**Keywords:** vitamin D receptor, polycystic ovary syndrome, polymorphism, meta-analysis, risk

## Abstract

**Objective:** Published studies have demonstrated a closer association between vitamin D receptor (VDR) gene polymorphisms and polycystic ovary syndrome (PCOS) risk, but the results were inconsistent. We therefore performed this meta-analysis to explore the precise associations between VDR gene polymorphisms and PCOS risk.

**Methods:** Five online electronic databases (PubMed, Embase, SCI index, CNKI and Wanfang) were searched. Odds ratios (ORs) with 95% confidence interval (CIs) were calculated to assess the association between VDR Fok I C/T (rs10735810), BsmI A/G (rs1544410), ApaI A/C (rs7975232), and TaqI T/C (rs731236) polymorphisms and PCOS risk. In addition, heterogeneity, accumulative/sensitivity analysis and publication bias were conducted to check the statistical power.

**Results:** Overall, 10 publications (31 independent case-control studies) involving 1,531 patients and 1,174 controls were identified. We found that the C mutation of ApaI A/C was a risk factor for PCOS (C vs. A: OR = 1.20, 95%CI = 1.06–1.35, *P* < 0.01, *I*^2^ = 29.7%; CC vs. AA: OR = 1.49, 95%CI = 1.17–1.91, *P* < 0.01, *I*^2^ = 0%; CC vs. AA+AC: OR = 1.36, 95%CI = 1.09–1.69, *P* = 0.01, *I*^2^ = 12.8%). Moreover, the BsmI A/G polymorphism also showed a dangerous risk for PCOS in Asian population (G vs. A: OR = 1.62, 95%CI = 1.24–2.11, *P* < 0.01, *I*^2^ = 0%; AG vs. AA: OR = 2.08, 95%CI = 1.26–3.20, *P* < 0.01, *I*^2^ = 0%; GG vs. AA: OR = 2.21, 95%CI = 1.29–3.77, *P* < 0.01, *I*^2^ = 0%; AG+GG vs. AA: OR = 2.12, 95%CI = 1.42–3.16, *P* < 0.01, *I*^2^ = 0%). In addition, no significant association of Fok I C/T, and TaqI T/C polymorphisms was observed.

**Conclusions:** In summary, our meta-analysis suggested that VDR gene polymorphisms contribute to PCOS development, especially in Asian populations.

## Introduction

Polycystic ovary syndrome (PCOS), characterized by clinical features including menstrual disorder, persistent anovulation, and polycystic ovaries, is one of the most common reproductive, endocrine, and metabolic disorder syndromes among women of reproductive age (Sirmans and Pate, 2013). Polycystic ovary syndrome (PCOS) has become a highly prevalent disorder that affects women in their reproductive age and contributes to multiple complications. According to the NIH 1990 criteria and/or Rotterdam 2003 criteria, the cumulative prevalence of PCOS was ~4–21% worldwide (Knochenhauer et al., 1998; Asuncion et al., 2000; Azziz et al., 2004). High prevalence and elevated risk of the development of type 2 diabetes mellitus (T2DM) and cardiovascular disease (CVD) were reported in women with PCOS (Repaci et al., 2011; Ollila et al., 2017). Moreover, long-term complications including the mental dysfunctions, such as mood and sleeping disorders, are also found. However, the precise etiology and underlying pathological mechanism of PCOS remain unclear.

Vitamin D, a steroid hormone, plays an important role in maintaining calcium homeostasis and promoting bone mineralization (Shen et al., 2013). Beyond these fundamental relationships, accumulating evidence indicates a close association of vitamin D status with the pathogenesis, signs and symptoms of PCOS (Wehr et al., 2009; Krul-Poel et al., 2013). A recent meta-analysis found significant differences in serum 25-hydroxyvitamin D, serum insulin, total cholesterol, triglycerides, and low-density lipoprotein cholesterol in patients with PCOS compared with that in healthy controls (Bacopoulou et al., 2017).

Vitamin D receptor (VDR) is widely distributed in several tissues of the female reproductive system (Kato, 2000). Vitamin D receptor (VDR) could mediate the biological responses of the 1α,25(OH)_2_D_3_ hormone, through generating a signal transduction complex with a heterodimer of 1α,25(OH)_2_D_3_-liganded VDR and unoccupied retinoid X receptor (RXR). Then, this transcriptional unit combines with the vitamin D response element (VDRE) in the promoter region of genes and regulates its actions through altering the transcriptional expression of target genes (Haussler et al., 2011). Single nucleotide polymorphisms (SNPs) are the most frequent nucleotide variations in the human genome. The VDR gene is located on chromosome 12q13.11, includes eight protein coding exons and one untranslated exon, and encodes a 427-amino-acid protein (Baker et al., 1988). To date, four most common VDR polymorphisms of FokI (rs10735810 C>T), BsmI (rs1544410 G>A), ApaI (rs7975232 G>T), and TaqI (rs731236 T>C) have been investigated to explore the association between VDR and PCOS susceptibility. However, the results were conflicting and inconclusive owing to the small sample size and limited statistical power. We therefore conducted this comprehensive meta-analysis to evaluate the association between the above polymorphisms and PCOS susceptibility precisely.

## Materials and Methods

This meta-analysis was conducted according to the guideline of Preferred Reporting Items for Systematic Reviews and Meta-Analyses (PRISMA) statement (Moher et al., 2009). All included data were based on published studies, and no ethical issues were involved.

### Search Strategy

Five online electronic databases (PubMed, Embase, SCI index, CNKI, and Wanfang) were searched with the following terms from their inception up to March 20, 2018: “vitamin D receptor,” “VDR,” “rs10735810,” “rs1544410,” “rs7975232,” “rs731236,” “polymorphism,” “variant,” “mutation” “polycystic ovary syndrome,” and “PCOS.” Some relevant references cited within retrieved articles were reviewed with manual searched.

The following search strategy was used:

#1 vitamin D receptor

#2 VDR

#3 rs10735810

#4 rs1544410

#5 rs7975232

#6 rs731236

#7 #1 OR #2 OR #3 OR #4 OR #5 OR #6

#8 polymorphism

#9 variant

#10 mutation

#11 #8 OR #9 OR #10

#12 polycystic ovary syndrome

#13 PCOS

#14 #12 OR #13

# 15 #7 AND #11 AND #14

### Eligibility Criteria

Studies were selected when they met the following criteria by two independent investigators (NYM and HYY): (1) the study followed a case-control design; (2) at least one polymorphisms of VDR gene was reported; (3) sufficient information about the distribution frequency of different polymorphism loci could be extracted to calculate the odds ratios (ORs) and 95% confidence intervals (CIs); (4) the most recent or largest sample sizes were selected when multiple publications were repeatedly reported with same team; and (5) the articles were written in English and Chinese.

### Data Extraction

All included studies were reviewed and extracted by two independent investigators (NYM and WYD). Disagreements and compared results were settled through discussion. The following information and data were extracted from included studies: the first author of each study, published year, study country or region where the study was conducted, ethnicity of research population, the source of the controls, the sample sizes of patients with PCOS and healthy controls, data of the frequency genotype of distribution, and the genotyping method.

### Risk Assessment of Bias Within Studies

All included studies in this meta-analysis were subject to make risk assessment of bias by two independent authors (JGB and BG) via the modified Newcastle-Ottawa Quality Assessment Scale (Niu et al., 2015). The score was based on five parameters (representativeness of cases, source of controls, Hardy-Weinberg equilibrium (HWE) in controls, genotyping examination and association assessment), with a maximum score of 11 points. Studies of at least a score of 8 were identified with a high quality (Table 1).

**Table 1:** Scale for quality evaluation.

**Criteria**	**Score**
**Representativeness of cases**	
Consecutive/randomly selected cases with clearly defined sampling frame Not consecutive/randomly selected case or without clearly defined sampling frame Not described	2 1 0
**Source of controls**	
Population-based Hospital-bases or healthy-bases Not described	2 1 0
**Hardy-weinberg equilibrium in controls**	
Hardy-weinberg equilibrium Hardy-weinberg disequilibrium Not available	2 1 0
**Genotyping examination**
Genotyping done under “blinded” condition and repeated again Genotyping done under “blinded” condition or repeated again Unblinded done or not mentioned and unrepeated	2 1 0
**Subjects**	
Number ≥300 Number < 300	1 0
**Association assessment**
Assess association between genotypes and PCOS with appropriate statistics and adjustment for confounders Assess association between genotypes and PCOS with appropriate statistics and without adjustment for confounders Inappropriate statistics used	2 1 0

### Statistical Analysis

Crude ORs with 95% CIs were calculated to examine the statistical power of the association between the VDR gene polymorphisms and PCOS risk. For example, four genetic models of Fok I C/T polymorphisms were calculated: allele contrast (T vs. C), co-dominant models (heterozygote model: CT vs. CC, homozygote model: TT vs. CC), dominant model (CT+TT vs. CC), and recessive model (TT vs. CC+CT) (Minelli et al., 2005; Lewis and Knight, 2012). Similar genetic models were also calculated with the others [BsmI A/G (rs1544410), ApaI A/C (rs7975232), TaqI T/C (rs731236)]. Subgroup analysis based on HWE status, ethnicity difference, control design and genotyping methods were performed to clarify the potential risk. Heterogeneity was investigated by *I*^2^ index which describes the percentage of variation among the included studies in the pooled analysis (Huedo-Medina et al., 2006). The fixed-effect model (Mantel-Haenszel method) was used when the *I*^2^ value was < 40% (Mantel and Haenszel, 1959). Otherwise, a random-effects model (DerSimonian and Laird method) was adopted (DerSimonian, 1996). Cumulative analyses were conducted to explore the tendency of the results whit the published studies were added. Sensitivity analyses were performed to investigate the stability of the results when each study was removed one at a time. Publication bias was assessed with the Egger's bias test and Begg's funnel plots (Begg and Mazumdar, 1994; Egger et al., 1997). Data analysis was conducted using STATA version 14.0 (Stata Corporation, College Station, TX, USA). *P* < 0.05 indicated a statistically significant difference.

## Results

### Study Characteristics

**Figure 1:**
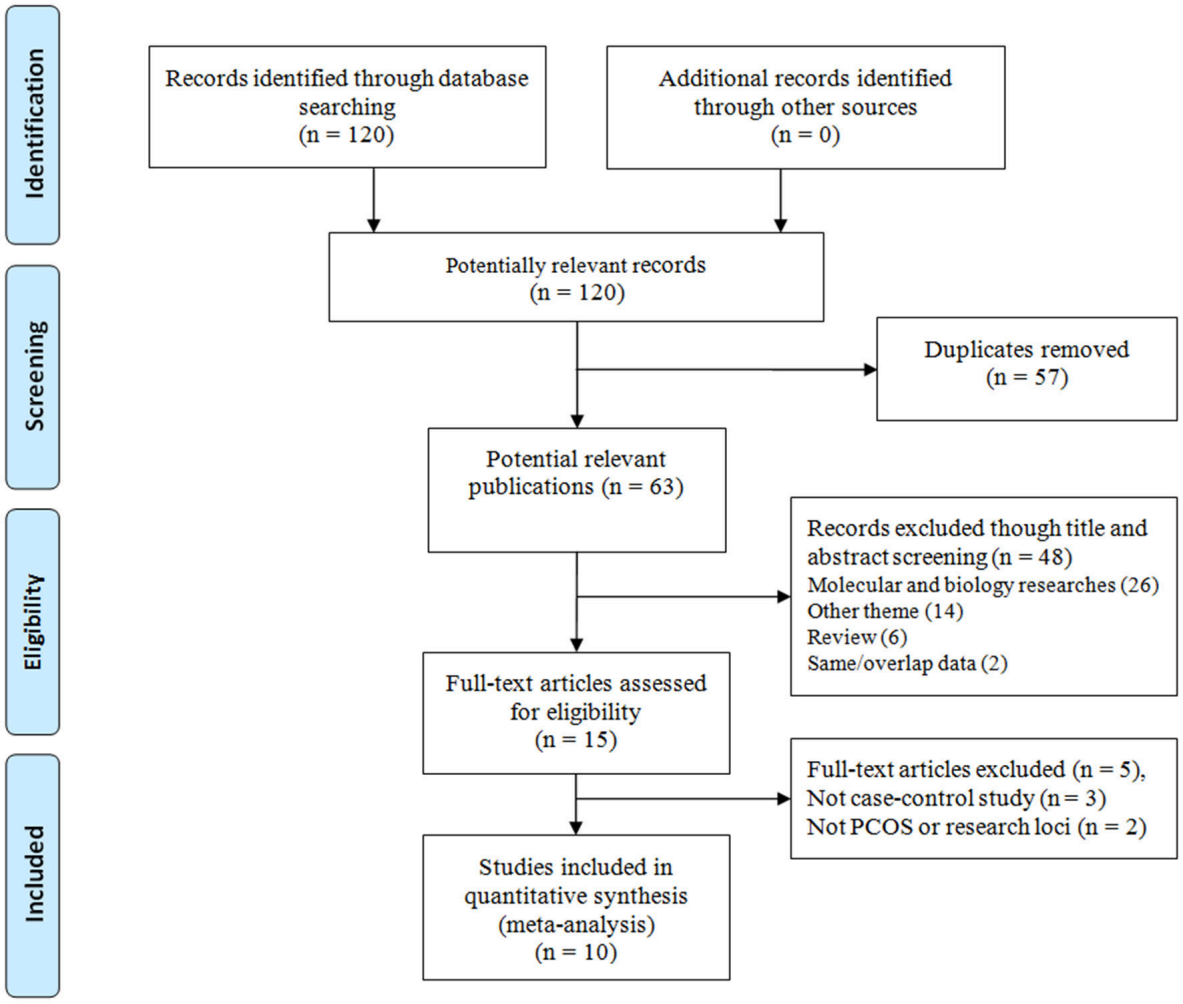
Flow diagram of the study selection process.

At first, 120 publications were identified through the systematic literature search. Three important steps according to the eligibility criteria were conducted to screen the selected studies were as follows: duplicate check, title and abstract check and text review. The selection of screening is presented in Figure 1. Finally, 10 studies were included in the meta-analysis with 1,531 patients with PCOS and 1,174 control individuals (Mahmoudi, 2009; Wehr et al., 2011; Bagheri et al., 2012, 2013; El-Shal et al., 2013; Dasgupta et al., 2015; Jedrzejuk et al., 2015; Mahmoudi et al., 2015; Cao and Tu, 2016; Siddamalla et al., 2018). The studies comprised seven case-control studies on FokI C/T (Mahmoudi, 2009; Wehr et al., 2011; Bagheri et al., 2012; Dasgupta et al., 2015; Jedrzejuk et al., 2015; Mahmoudi et al., 2015; Cao and Tu, 2016), seven case-control studies on BsmI A/G (Mahmoudi, 2009; Wehr et al., 2011; Bagheri et al., 2012; Jedrzejuk et al., 2015; Mahmoudi et al., 2015; Siddamalla et al., 2018), eight case-control studies on ApaI A/C (Mahmoudi, 2009; Wehr et al., 2011; El-Shal et al., 2013; Dasgupta et al., 2015; Jedrzejuk et al., 2015; Mahmoudi et al., 2015; Cao and Tu, 2016; Siddamalla et al., 2018), and nine case-control studies on TaqI T/C (Mahmoudi, 2009; Wehr et al., 2011; Bagheri et al., 2013; El-Shal et al., 2013; Dasgupta et al., 2015; Jedrzejuk et al., 2015; Mahmoudi et al., 2015; Cao and Tu, 2016; Siddamalla et al., 2018), respectively. Furthermore, three publications involved the Asians (Dasgupta et al., 2015; Cao and Tu, 2016; Siddamalla et al., 2018), and seven studies involved Caucasians (Mahmoudi, 2009; Wehr et al., 2011; Bagheri et al., 2012, 2013; El-Shal et al., 2013; Jedrzejuk et al., 2015; Mahmoudi et al., 2015). In the control groups, there are two case-control studies in BsmI A/G (Mahmoudi, 2009; Siddamalla et al., 2018), three case-control studies in ApaI A/C (Wehr et al., 2011; Dasgupta et al., 2015; Siddamalla et al., 2018) and two case-control studies in TaqI T/C (Cao and Tu, 2016; Siddamalla et al., 2018) polymorphisms deviated from the HWE. The main characteristics of the selected studies are shown in Table 2.

**Table 2:** Characteristics of included studies on vitamin D receptor gene polymorphisms and polycystic ovary syndrome risk.

**References**	**Country/region**	**Racial**	**Source of controls**	**Case**	**Control**	**Genotype distribution**						**Genotyping methods**	***P* for HWE**	**MAF in control**	**NOS**
						**Case**			**Control**						
						**Fok I C/T (rs10735810)**									
						**CC**	**CT**	**TT**	**CC**	**CT**	**TT**				
Mahmoudi, 2009	Iran	Caucasian	Hospital-B	162	162	83	67	12	96	59	7	PCR-RFLP	0.58	0.23	8
Wehr et al., 2011	Austria	Caucasian	Hospital-B	538	135	215	241	82	53	60	22	Genotyping assay	0.47	0.39	8
Bagheri et al., 2012	Iran	Caucasian	Healthy-B	46	46	22	20	4	29	15	2	PCR-RFLP	0.97	0.21	7
Jedrzejuk et al., 2015	Poland	Caucasian	Healthy-B	90	98	28	51	11	23	50	25	PCR- SNaPshot	0.84	0.51	7
Dasgupta et al., 2015	India	Asian	Healthy-B	250	250	155	85	10	153	82	15	PCR-RFLP	0.37	0.22	8
Mahmoudi et al., 2015	Iran	Caucasian	Healthy-B	35	35	16	17	2	24	10	1	PCR-RFLP	0.97	0.17	8
Cao and Tu, 2016	China	Asian	Hospital-B	120	120	70	40	10	65	45	10	PCR-RFLP	0.60	0.27	7
						**BsmI A/G (rs1544410)**									
						**AA**	**AG**	**GG**	**AA**	**AG**	**GG**				
Mahmoudi, 2009	Iran	Caucasian	Hospital-B	162	162	24	85	53	18	91	53	PCR-RFLP	0.02	0.61	8
Wehr et al., 2011	Austria	Caucasian	Hospital-B	537	137	77	244	216	22	66	49	Genotyping assay	0.98	0.60	8
Bagheri et al., 2012	Iran	Caucasian	Healthy-B	46	46	15	27	4	20	24	2	PCR-RFLP	0.12	0.30	7
Jedrzejuk et al., 2015	Poland	Caucasian	Healthy-B	90	98	14	45	31	13	42	43	PCR- SNaPshot	0.59	0.65	7
Mahmoudi et al., 2015	Iran	Caucasian	Healthy-B	35	35	10	12	13	5	23	7	PCR-RFLP	0.06	0.53	8
Cao and Tu, 2016	China	Asian	Hospital-B	120	120	23	60	37	40	55	25	PCR-RFLP	0.45	0.44	7
Siddamalla et al., 2018	India	Asian	Hospital-B	95	130	35	45	15	72	41	17	PCR-RFLP	0.01	0.29	7
						**ApaI A/C (rs7975232)**									
						**AA**	**AC**	**CC**	**AA**	**AC**	**CC**				
Mahmoudi, 2009	Iran	Caucasian	Hospital-B	162	162	58	68	36	49	90	23	PCR-RFLP	0.07	0.42	8
Wehr et al., 2011	Austria	Caucasian	Hospital-B	543	145	142	274	127	48	60	37	Genotyping assay	0.04	0.46	7
El-Shal et al., 2013	Egypt	Caucasian	Healthy-B	150	150	63	65	22	68	64	18	PCR-RFLP	0.62	0.33	8
Jedrzejuk et al., 2015	Poland	Caucasian	Healthy-B	90	98	19	52	19	32	49	17	PCR- SNaPshot	0.81	0.42	7
Dasgupta et al., 2015	India	Asian	Healthy-B	250	250	117	120	13	122	116	12	PCR-RFLP	0.02	0.28	7
Mahmoudi et al., 2015	Iran	Caucasian	Healthy-B	35	35	15	11	9	8	21	6	PCR-RFLP	0.23	0.47	8
Cao and Tu, 2016	China	Asian	Hospital-B	120	120	22	58	40	39	55	26	PCR-RFLP	0.43	0.45	7
Siddamalla et al., 2018	India	Asian	Hospital-B	95	130	42	21	32	70	35	25	PCR-RFLP	< 0.01	0.33	7
						**TaqI T/C (rs731236)**									
						**TT**	**TC**	**CC**	**TT**	**TC**	**CC**				
Mahmoudi, 2009	Iran	Caucasian	Hospital-B	162	162	71	71	20	72	76	14	PCR-RFLP	0.33	0.32	8
Wehr et al., 2011	Austria	Caucasian	Hospital-B	536	137	226	238	72	49	65	23	Genotyping assay	0.85	0.41	8
Bagheri et al., 2013	Iran	Caucasian	Healthy-B	38	38	16	14	8	17	19	2	PCR-RFLP	0.26	0.30	7
El-Shal et al., 2013	Egypt	Caucasian	Healthy-B	150	150	40	74	36	69	61	20	PCR-RFLP	0.27	0.34	8
Jedrzejuk et al., 2015	Poland	Caucasian	Healthy-B	90	98	37	45	8	49	37	12	PCR- SNaPshot	0.24	0.31	7
Dasgupta et al., 2015	India	Asian	Healthy-B	250	250	112	91	47	109	104	37	PCR-RFLP	0.14	0.36	8
Mahmoudi et al., 2015	Iran	Caucasian	Healthy-B	35	35	15	14	6	15	16	4	PCR-RFLP	0.93	0.34	8
Cao and Tu, 2016	China	Asian	Hospital-B	120	120	57	52	11	40	72	8	PCR-RFLP	< 0.01	0.37	6
Siddamalla et al., 2018	India	Asian	Hospital-B	95	130	40	31	24	71	42	17	PCR-RFLP	0.01	0.29	7

*HWE in control. MAF, Minor allele frequency in control group; Hospital-B, Hospital-based control; Healthy-B, Healthy-based control*.

### Quantitative Analysis

#### Fok I C/T Locus and PCOS Risk

**Figure 2:**
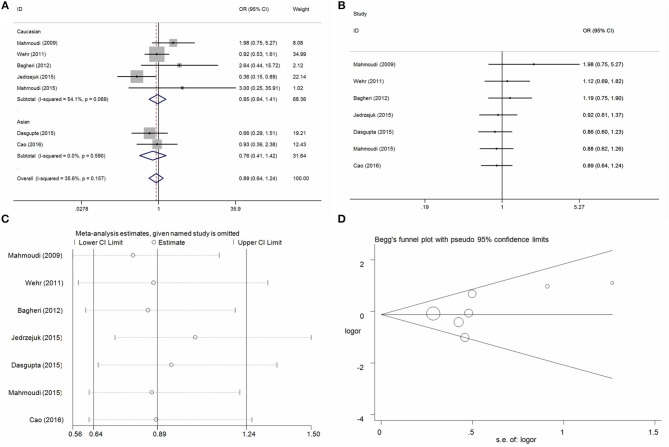
Statistical analysis of the association between VDR Fok I C/T polymorphism and PCOS risk in the TT vs. CC model. **(A)** ORs and 95% CIs; **(B)** cumulative analysis; **(C)** sensitivity analysis; **(D)** publication bias.

Seven case-control studies with 1,241 PCOS cases and 846 control individuals were identified with regard to the association between Fok I C/T locus and PCOS risk. Overall, the pool analysis did not find any significant association between this locus on PCOS risk in five genetic models (T vs. C: OR = 1.04, 95%CI = 0.83–1.30, *P* = 0.77, *I*^2^ = 53.2%; CT vs. CC: OR = 1.08, 95%CI = 0.89–1.32, *P* = 0.40, *I*^2^ = 7.0%; TT vs. CC: OR = 0.89, 95%CI = 0.64–1.25, *P* = 0.50, *I*^2^ = 35.6%; CT+TT vs. CC: OR = 1.06, 95%CI = 0.88–1.27, *P* = 0.56, *I*^2^ = 36.4%; TT vs. CC+CT: OR = 0.86, 95%CI = 0.63–1.18, *P* = 0.34, *I*^2^ = 23.6%) (Table 3, Figure 2A for TT vs. CC model). Heterogeneity was only indentified in allele contrast and the meta-regression analyses did not find any distinct factors that contributed to the heterogeneity. Furthermore, no significant association was identified in stratified analysis of HWE status, ethnicity difference, and control design and genotyping methods (Table 3). Cumulative analyses by publication date showing the negative results according to the new studies were added (Figure 2B for TT vs. CC model). Sensitivity analysis presented a consistent tendency of negative results without any apparent changes (Figure 2C for TT vs. CC model). Publication bias was assessed using the Egger's bias test and Begg's funnel plot tests, and no significant asymmetrical evidence was found (T vs. C: *P* = 0.23; CT vs. CC: *P* = 0.20; TT vs. CC: *P* = 0.33; CT+TT vs. CC: *P* = 0. 27; TT vs. CC+CT: *P* = 0.37) (Figure 2D for TT vs. CC model).

**Table 3:** Summary ORs and 95% CI of vitamin D receptor gene polymorphisms and polycystic ovary syndrome risk.

**Locus**	N^*^	**OR**	**95% CI**	***P***	***I*^**2**^**	**OR**	**95% CI**	***P***	***I*^**2**^**	**OR**	**95% CI**	***P***	***I*^**2**^**	**OR**	**95% CI**	***P***	***I*^**2**^**	**OR**	**95% CI**	***P***	***I*^**2**^**
**Fok I C/T (rs10735810)**		**T vs. C**				**CT vs. CC**				**TT vs. CC**				**CT+TT vs. CC**				**TT vs. CC+CT**			
Total	7	1.04	0.83–1.30	0.77	53.2	1.08	0.89–1.32	0.42	7.0	0.89	0.64–1.25	0.50	35.6	1.06	0.88–1.27	0.56	36.4	0.86	0.63–1.18	0.34	23.6
**ETHNICITY**																					
Caucasian	5	1.14	0.80–1.60	0.47	66.6	1.18	0.92–1.53	0.20	19.3	1.07	0.52–2.19	0.86	54.1	1.22	0.84–1.77	0.31	49.5	0.96	0.53–1.76	0.91	45.0
Asian	2	0.91	0.72–1.16	0.46	0	0.95	0.70–1.30	0.77	0	0.77	0.41–1.43	0.40	0	0.92	0.69–1.24	0.60	0	0.79	0.43–1.45	0.44	0
**DESIGN**																					
Hospital-B	3	1.04	0.86–1.27	0.65	28.1	1.04	0.80–1.36	0.75	0.0	1.07	0.70–1.56	0.76	0	1.05	0.82–1.36	0.68	15.9	1.06	0.71–1.59	0.78	0
Healthy-B	4	1.08	.0.69–1.69	0.72	68.7	1.14	0.85–1.52	0.39	33.8	0.78	0.33–1.83	0.56	45.1	1.19	0.72–1.97	0.50	57.5	0.62	0.37–1.03	0.07	23.7
**GENOTYPING**																					
PCR-RFLP	5	1.17	0.89–1.54	0.26	45.9	1.15	0.91–1.46	0.27	26.6	1.14	0.70–1.84	0.59	11.6	1.22	0.88–1.68	0.24	42.3	1.09	0.68–1.75	0.72	0
Other	2	0.82	0.56–1.19	0.30	57.7	0.95	0.67–1.35	0.76	0	0.62	0.25–1.53	0.30	66.6	0.88	0.63–1.23	0.47	0	0.65	0.29–1.44	0.28	66.5
**BsmI A/G (rs1544410)**		**G vs. A**				**AG vs. AA**				**GG vs. AA**				**AG+GG vs. AA**				**GG vs. AA+AG**			
Total	7	1.17	0.95–1.45	0.14	49.6	1.15	0.75–1.78	0.52	59.9	1.28	0.95–1.74	0.11	35.7	1.22	0.82–1.81	0.34	57.1	1.16	0.93–1.45	0.18	19.8
HWE-yes	5	1.17	0.90–1.51	0.24	45.2	1.10	0.67–1.82	0.70	50.8	1.38	0.96–2.00	0.09	36.9	1.19	0.76–1.89	0.48	47.5	1.26	0.84–1.89	0.27	42.2
HWE-no	2	1.20	0.70–2.07	0.50	78.2	1.27	0.40–4.01	0.68	84.7	1.15	0.48–2.72	0.76	61.2	1.26	0.43–3.64	0.67	84.1	1.06	0.72–1.58	0.16	0
**ETHNICITY**																					
Caucasian	5	1.02	0.86–1.21	0.84	0.0	0.90	0.64–1.26	0.54	31.4	0.99	0.68–1.44	0.95	0.0	0.94	0.68–1.30	0.72	11.7	1.08	0.84–1.39	0.57	27.2
Asian	2	1.62	1.24–2.11	< 0.01	0.0	2.08	1.26–3.20	< 0.01	0	2.21	1.29–3.77	< 0.01	0.0	2.12	1.42–3.16	< 0.01	0	1.51	0.95–2.39	0.08	0
**DESIGN**																					
Hospital-B	4	1.26	0.97–1.64	0.08	60.1	1.35	0.81–2.24	0.25	64.5	1.43	0.87–2.35	0.16	51.3	1.41	0.86–2.29	0.17	65.5	1.22	0.95–1.57	0.12	0
Healthy-B	3	0.96	0.71–1.31	0.82	20.8	0.82	0.33–2.00	0.66	59.8	0.90	0.45–1.78	0.76	0	0.90	0.45–1.80	0.76	40.0	1.28	0.49–3.30	0.62	58.9
**GENOTYPING**																					
PCR-RFLP	5	1.29	0.99–1.99	0.06	46.3	1.17	0.62–2.20	0.63	71.6	1.49	0.85–2.60	0.16	40.7	1.29	0.74–2.25	0.37	66.9	1.30	0.97–1.80	0.07	0
Other	2	0.98	0.68–1.41	0.89	55.3	1.04	0.65–1.65	0.87	0	1.04	0.65–1.69	0.86	28.1	1.04	0.67–1.61	0.85	0	0.94	0.53–1.66	0.84	62.2
**ApaI A/C (rs7975232)**		**C vs. A**				**AC vs. AA**				**CC vs. AA**				**AC+CC vs. AA**				**CC vs. AA+AC**			
Total	8	1.20	1.06–1.35	< 0.01	29.7	1.10	0.80–1.49	0.56	59.7	1.49	1.17–1.91	< 0.01	0	1.21	0.94–1.57	0.15	51.3	1.36	1.09–1.69	0.01	12.8
HWE-yes	5	1.22	1.03–1.45	0.02	32.6	1.00	0.58–1.72	0.99	72.6	1.61	1.13–2.27	0.01	0	1.14	0.70–1.83	0.60	68.8	1.55	1.15–2.10	< 0.01	0
HWE-no	3	1.21	0.95–1.54	0.13	49.0	1.21	0.94–1.56	0.14	0	1.38	0.97–1.97	0.07	18.1	1.26	1.00–1.59	0.06	0	1.25	0.71–2.20	0.44	62.7
**ETHNICITY**																					
Caucasian	5	1.11	0.95–1.30	0.18	0	0.98	0.60–1.62	0.95	72.6	1.28	0.94–1.74	0.12	0	1.07	0.74–1.57	0.71	58.0	1.20	0.91–1.57	0.20	0
Asian	3	1.41	1.02–1.95	0.04	63.9	1.19	0.90–1.57	0.23	18.9	1.97	1.30–2.97	< 0.01	22.8	1.42	0.96–2.10	0.08	48.6	1.72	1.19–2.50	< 0.01	0
**DESIGN**																					
Hospital-B	4	1.31	1.02–1.67	0.03	55.8	1.16	0.71–1.89	0.56	68.9	1.59	1.17–2.15	< 0.01	38.1	1.33	0.90–1.97	0.16	61.1	1.50	0.98–2.30	0.06	59.9
Healthy-B	4	1.11	0.93–1.34	0.24	0	1.03	0.64–1.64	0.91	60.5	1.31	0.85–2.02	0.22	0	1.10	0.74–1.62	0.64	49.9	1.26	0.85–1.87	0.25	0
**GENOTYPING**																					
PCR-RFLP	5	1.22	1.00–1.49	0.05	45.4	0.95	0.66–1.36	0.78	58.5	1.60	1.18–2.16	< 0.01	3.8	1.10	0.80–1.54	0.52	57.8	1.63	1.24–2.15	< 0.01	0
Other	2	1.17	0.94–1.46	0.16	0	1.61	1.12–2.32	0.01	0	1.30	0.85–2.00	0.22	0	1.50	1.07–2.10	0.02	0	0.98	0.68–1.41	0.89	0
**TaqI T/C (rs731236)**		**C vs. T**				**TC vs. TT**				**CC vs. TT**				**TC+ CC vs. TT**				**CC vs. TT+ TC**			
Total	9	1.16	0.94–1.44	0.18	66.5	1.00	0.75–1.34	0.99	58.5	1.44	0.97–2.15	0.07	54.2	1.10	0.82–1.48	0.52	65.4	1.37	1.09–1.74	0.01	38.3
HWE-yes	7	1.16	0.93–1.46	0.19	61.3	1.07	0.79–1.45	0.66	51.2	1.39	0.87–2.22	0.17	57.8	1.15	0.85–1.56	0.38	58.1	1.31	0.90–1.90	0.16	43.4
HWE-no	2	1.15	0.52–2.53	0.73	88.0	0.81	0.32–2.05	0.65	81.1	1.65	0.65–4.18	0.29	56.3	0.95	0.33–2.80	0.93	87.9	1.91	1.09–3.33	0.02	0
**ETHNICITY**																					
Caucasian	6	1.20	0.89–1.60	0.23	67.2	1.13	0.79–1.63	0.50	54.2	1.46	0.79–2.71	0.23	64.8	1.20	0.82–1.76	0.34	62.8	1.33	0.81–2.17	0.26	52.5
Asian	3	1.11	0.74–1.67	0.60	76.4	0.82	0.51–1.32	0.41	63.0	1.45	0.99–2.12	0.06	36.9	0.95	0.55–1.65	0.86	75.9	1.55	1.08–2.22	0.02	0
**DESIGN**																					
Hospital-B	4	1.03	0.74–1.44	0.85	74.5	0.83	0.59–1.17	0.29	48.0	1.23	0.66–2.27	0.52	63.5	0.91	0.60–1.38	0.66	68.0	1.33	0.79–2.22	0.28	54.0
Healthy-B	5	1.31	1.00–1.70	0.05	48.0	1.21	0.78–1.87	0.40	57.9	1.70	0.98–2.96	0.06	46.0	1.33	0.89–1.98	0.16	56.4	1.51	1.09–2.08	0.01	30.8
**GENOTYPING**																					
PCR-RFLP	7	1.25	0.97–1.60	0.08	64.5	0.98	0.69–1.40	0.91	61.8	1.74	1.30–2.33	< 0.01	27.3	1.14	0.79–1.63	0.50	68.5	1.68	1.28–2.22	< 0.01	0
Other	2	0.89	0.71–1.13	0.34	38.9	1.09	0.55–2.17	0.81	71.8	0.73	0.44–1.19	0.20	0	1.01	0.55–1.86	0.98	68.1	0.75	0.48–1.18	0.22	0

* *Numbers of comparisons*.

*Hospital-B, Hospital-based control; Healthy-B, Healthy-based control; RT-PCR, Real-time PCR*.

#### BsmI A/G Locus and PCOS Risk

**Figure 3:**
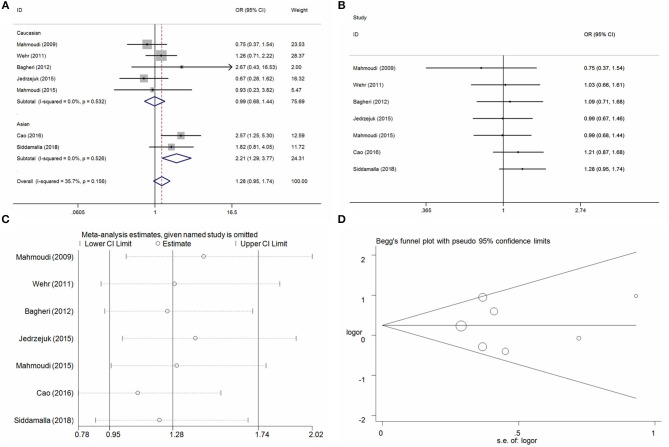
Statistical analysis of the association between VDR BsmI A/G polymorphism and PCOS risk in the GG vs. AA model. **(A)** ORs and 95% CIs; **(B)** cumulative analysis; **(C)** sensitivity analysis; **(D)** publication bias.

Seven case-control studies with 1,085 PCOS cases and 728 control individuals were identified on the association between BsmI A/G locus and PCOS risk. Overall, the pool analysis did not find any significant association between this locus on PCOS risk in five genetic models (G vs. A: OR = 1.17, 95%CI = 0.95–1.45, *P* = 0.14, *I*^2^ = 49.6%; AG vs. AA: OR = 1.15, 95%CI = 0.75–1.78, *P* = 0.52, *I*^2^ = 59.9%; GG vs. AA: OR = 1.28, 95%CI = 0.95–1.74, *P* = 0.11, *I*^2^ = 35.7%; AG+GG vs. AA: OR = 1.22, 95%CI = 0.82–1.81, *P* = 0.34, *I*^2^ = 57.1%; GG vs. AA+AG: OR = 1.16, 95%CI = 0.93–1.45, *P* = 0.18, *I*^2^ = 19.8%) (Table 3, Figure 3A for GG vs. AA model). Heterogeneity was observed in allele contrast, heterozygote model and dominant model. Meta-regression analyses were conducted, and the results indicated that the ethnicity diversity maybe the critical factors contributing to the existed heterogeneity (G vs. A: *P*_ethnicity_ = 0.04; AG vs. AA: *P*_ethnicity_ = 0.04; AG+GG vs. AA: *P*_ethnicity_ = 0.03). In addition, the subgroup of ethnicity proved that the heterogeneity was alleviated in the Asian and Caucasian subgroups apparently. Furthermore, the subgroup analyses based on ethnicity presented an increased risk in Asian populations in some genetic models (G vs. A: OR = 1.62, 95%CI = 1.24–2.11, *P* < 0.01, *I*^2^ = 0%; AG vs. AA: OR = 2.08, 95%CI = 1.26–3.20, *P* < 0.01, *I*^2^ = 0%; GG vs. AA: OR = 2.21, 95%CI = 1.29–3.77, *P* < 0.01, *I*^2^ = 0%; AG+GG vs. AA: OR = 2.12, 95%CI = 1.42–3.16, *P* < 0.01, *I*^2^ = 0%). Cumulative analyses by publication date were conducted and indicated apparent consistence and stability of pool results (Figure 3B for GG vs. AA model). Sensitivity analysis was conducted and indicated some changes of results in allele contrast, homozygote, and recessive models without the publication by Jedrzejuk et al. (2015) (Figure 3C for GG vs. AA model). Publication bias was assessed using the Egger bias test and a Begg funnel plot test, and no significant asymmetrical evidence was found (T vs. C: *P* = 0.82; CT vs. CC: *P* = 0.17; TT vs. CC: *P* = 0.94; CT+TT vs. CC: *P* = 0.19; TT vs. CC+CT: *P* = 0.36) (Figure 3D for GG vs. AA model).

### ApaI A/C Locus and PCOS Risk

**Figure 4:**
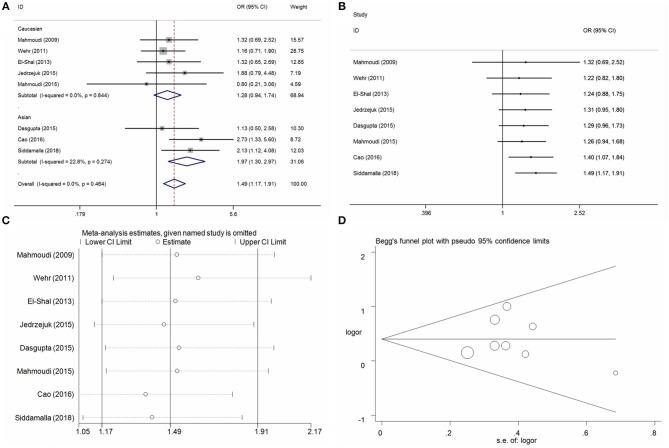
Statistical analysis of the association between VDR ApaI A/C polymorphism and PCOS risk in the CC vs. AA model. **(A)** ORs and 95% CIs; **(B)** cumulative analysis; **(C)** sensitivity analysis; **(D)** publication bias.

Eight case-control studies with 1,445 cases and 1,090 controls individuals were identified on the association between ApaI A/C locus and PCOS risk. Overall, significant association of increased risk were observed in three genetic models (C vs. A: OR = 1.20, 95%CI = 1.06–1.35, *P* = 0.01, *I*^2^ = 29.7%; CC vs. AA: OR = 1.49, 95%CI = 1.17–1.91, *P* < 0.01, *I*^2^ = 0%; CC vs. AA+AC: OR = 1.36, 95%CI = 1.09–1.69, *P* = 0.01, *I*^2^ = 12.8%) (Table 3, Figure 4A for CC vs. AA model). Heterogeneity was observed in heterozygote model (AC vs. AA) and dominant model (AC+CC vs. AA), and the meta-regression analyses did not find any distinct factors that contributed to the heterogeneity. Subgroup analyses by ethnicity presented an increased risk in Asian populations in the genetic models mentioned (C vs. A: OR = 1.22, 95%CI = 1.03–1.45, *P* = 0.02, *I*^2^ = 32.6%; CC vs. AA: OR = 1.61, 95%CI = 1.13–2.27, *P* < 0.01, *I*^2^ = 0%; CC vs. AA+AC: OR = 1.55, 95%CI = 1.15–2.10, *P* = 0.01, *I*^2^ = 0%). Moreover, the same significant PCOS risk was observed in some genetic models in these subgroups of HWE-yes, hospital control and genotyping groups (Table 3). Cumulative analyses demonstrated a significant alteration when the study of Cao and Tu (2016) was added in 2016 (Table 2, Figure 4B for CC vs. AA model). Sensitivity analysis was conducted in every genetic model and did not indicate some apparent changes except for the dominant modes (Figure 4C for CC vs. AA model). Publication bias was assessed using the Egger bias test and a Begg's funnel plot test, and no significant asymmetrical evidence was found (C vs. A: *P* = 0.74; AC vs. AA: *P* = 0.55; CC vs. AA: *P* = 0.97; AC+CC vs. AA: P = 0.86; CC vs. AA+AC: *P* = 0.37) (Figure 4D for CC vs. AA model).

### TaqI T/C Locus and PCOS Risk

**Figure 5:**
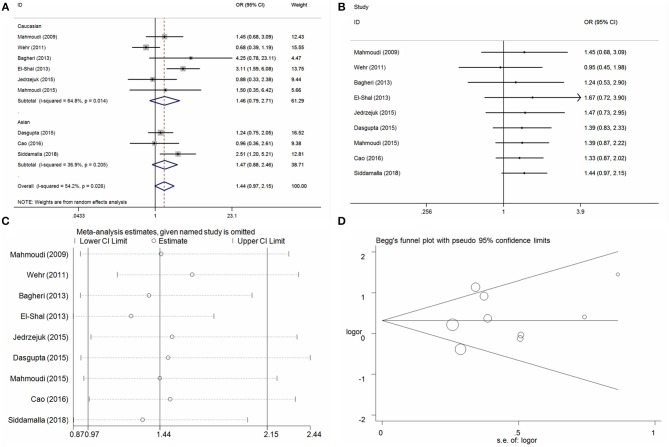
Statistical analysis of the association between VDR TaqI T/C polymorphism and PCOS risk in the CC vs. TT model. **(A)** ORs and 95% CIs; **(B)** cumulative analysis; **(C)** sensitivity analysis; **(D)** publication bias.

Nine case-control studies with 1,476 cases and 1,120 controls individuals were identified on the association between TaqI T/C locus and PCOS risk. Overall, the increased risk was observed only in the recessive model (CC vs. TT+TC: OR = 1.37, 95%CI = 1.09-1.74, *P* = 0.01, *I*^2^ = 38.3%) (Table 3, Figure 5A for CC vs. TT model). Heterogeneities were identified in the allele contrast (C vs. T), heterozygote model (TC vs. TT), homozygote (CC vs. TT), and dominant model (TC+CC vs. TT). Meta-regression analyses only found that the genotyping methods contributed to the existing heterogeneity in the dominant model, but not in other models. Subgroup analyses revealed an increased PCOS risks in Asian populations (CC vs. TT+TC: OR = 1.55, 95%CI = 1.08-2.22, *P* = 0.02, *I*^2^ = 0%) and other subgroups in the recessive model (Table 3). Cumulative analyses by publication date demonstrated a negative association except for the recessive model (Figure 5B for CC vs. TT model). Sensitivity analysis indicated some slight alterations when the studies of Wehr et al. (2011) and El-Shal et al. (2013) were deleted in the homozygote and recessive models, respectively (Figure 5C for CC vs. TT model). Publication bias was assessed using the Egger bias test and a Begg funnel plot test, and no significant asymmetrical evidence was found (C vs. T: *P* = 0.48; TC vs. TT: *P* = 0.74; CC vs. TT: *P* = 0.49; TC+CC vs. TT: *P* = 0.62; CC vs. TT+TC: *P* = 0.38) (Figure 5D for CC vs. TT model).

## Discussion

To date, the pathogenesis and etiology of PCOS have remained unknown. The complex gene-gene and gene-environment interactions have been reported to be an important risk for PCOS development. Consistent epidemiologic evidence demonstrated that PCOS always suffered a series of complications, comprising hyperandrogenism, oligo-anovulation, insulin resistance and associated metabolic abnormalities.

Many studies proved that a dysregulated vitamin D serum level is closely related to PCOS occurrence. In addition, the vitamin D supplementation therapy would decreases fasting plasma glucose, serum insulin concentrations, and homeostasis model assessment-estimated insulin resistance (HOMA-IR) (Asemi et al., 2015; Foroozanfard et al., 2017). All these evidences suggested that vitamin D disorder is associated with multiple metabolic risks in women with PCOS.

Noteworthily, the 1α,25(OH)_2_D_3_ is an important active form of vitamin D, it is mediated by the vitamin D receptor [1α,25(OH)_2_D_3_ receptor, VDR] (Yoshizawa et al., 1997). Vitamin D receptor (VDR) is a DNA-binding transcription factor, combined with a heterodimer of the 1a,25(OH)_2_D_3_-ligand VDR and unoccupied RXR to generate an active signal transduction complex (Haussler et al., 2011). To date, several functional SNP loci reported in these polymorphisms presented an increased susceptibility of various diseases (Valdivielso and Fernandez, 2006), such as multiple cancers (Vidigal et al., 2017), diabetes mellitus (Yu et al., 2017), rheumatoid arthritis (Tizaoui et al., 2014), and cardiocerebrovascular disease (Moradi et al., 2017).

In 2009, Mahmoudi et al. published the first case-control study to explore the association between the above four polymorphisms and PCOS susceptibility in the Iranian population, and the results suggested that these individuals with an CC genotype have an increased risk for PCOS compared with the AA genotype. Since then, a series of case-control studies was conducted to evaluate the association between the vitamin D polymorphisms and PCOS susceptibility, but some controversies arose and bewildered us completely. In 2017, Reis et al. published a system review on vitamin D polymorphisms and PCOS with most literature on this theme (Reis et al., 2017). However, the synthesis and calculation of all selected data were not conducted. We also made a comprehensive understanding of all the studies but failed to draw a clear conclusion. Thus, we conducted the meta-analysis to investigate the precise relationships between VDR Fok I C/T, BsmI A/G, ApaI A/C, and TaqI T/C polymorphisms and PCOS risk based on 10 published case-control studies.

In the current meta-analysis, the pooled results indicated some significant association between ApaI A/C polymorphism and PCOS susceptibility in allele contrast, homozygote genotype and recessive models, presenting 1.20-, 1.49- and 1.36-fold high risk for PCOS. Furthermore, an increased PCOS risk was observed in the subgroup analysis of the HWE-yes group and hospital based group, especially in the Asian group. In BsmI A/G polymorphism, only some increased PCOS risks were observed in the Asians based on ethnic diversity. These pieces of evidence demonstrated that the ethnicity differences may play an important role, contributing to the varying PCOS susceptibility among the Asian and Caucasian races. In addition, no signification association was observed in TaqI T/C and Fok I C/T polymorphisms for PCOS risk, except for a few scattered cases of increased PCOS risk in the former in the recessive models.

The restriction fragment length polymorphism sties of BsmI and ApaI are located in the intron (between exons 8 and 9), and the TaqI polymorphism was located in exon 9 (Zmuda et al., 2000). They are all located near the 3'-untranslated region of the VDR gene, which was suggested to be involved in the regulation of gene expression by modulating mRNA stability (Zmuda et al., 2000; Ogunkolade et al., 2002). In this meta-analysis, some increased and significant risks were observed in the above three polymorphism, indicating that the potential synergism among these polymorphisms would play an important role for PCOS occurrence. Regrettably, this hypothesis could not be verified without valid haplotype data to assess interaction between the adjacent polymorphism loci with all included studies. FokI polymorphism located in exon 2, resulting in a incorporation VDR protein production in the NH2 terminal, which was suggested to influence the transcriptional activity of VDR gene combined with the modulation of transcription factor IIB (Jurutka et al., 2000; Whitfield et al., 2001). Some publications indicated that this polymorphism would regulate the expression of mRNA and contribute to susceptibility to various diseases (Arai et al., 1997; Colombini et al., 2014), but no significant association between FokI polymorphism and PCOS was found based on the current meta-analysis. So, all these evidences indicated that there were a causation between the mutation of the above SNPs located in VDR gene and PCOS occurrence (Hill, 1965).

To our knowledge, this is the first meta-analysis to assess the association between VDR polymorphisms and PCOS risk. Some advantages were presented in this meta-analysis compared with the published case-control studies: First, all case-control studies published on the four polymorphisms were considered, and the risk assessment of bias within studies would enhance the statistical power and help understand the association between VDR polymorphisms and PCOS risk. Second, a stratified analysis based on ethnic diversity, control design, and genotyping methods was conducted to explore the potential relationships that were modulated under these subgroup biologic factors. Third, a scientific retrieval strategy and rigorous methodology were used, including cumulative analyses and sensitivity analyses. Publication bias was also used to guarantee the stability and credibility of the conclusions of the analysis.

However, there were some limitations of this study, which should be pointed out. First, only 10 publications were included in this present meta-analysis. The studies and sample size of each polymorphic locus were limited, and the pooled results and subgroup analysis could not reveal the reality association between VDR polymorphisms and PCOS susceptibility. Second, interactive risk factors, such as living habits, diet, age, and family history were not adjusted in this meta-analysis due to data deficiency. Third, included studies were written only in English and Chinese, and the included subjects were mostly Asian and Caucasian populations. Therefore, the results of this meta-analysis cannot represent all ethnic populations, and the application of the conclusions was restricted. Fourth, all examined polymorphisms were assessed separately, and the gene-gene interactions especially the haplotype analyses were not assessed due to the insufficient data.

In conclusion, this meta-analysis suggests that VDR gene polymorphisms play an important role in PCOS development, especially on the ApaI A/C and BsmI A/G among the Asian populations. Further case-control studies on various ethnic populations with a larger sample size are need to verify the current conclusions in the future.

## Author Contributions

Y-MN, Y-DW, and Y-YH conceived the study. Y-MN and Y-DW searched the databases and extracted the data. X-FL, G-BJ, and GB analyzed the data. Y-MN, Y-YH, and H-BC wrote the draft of the paper. Y-DW and MS reviewed the manuscript. All the authors approved the final manuscript.

### Conflict of Interest Statement

The authors declare that the research was conducted in the absence of any commercial or financial relationships that could be construed as a potential conflict of interest.
